# Influence of Innovative Post-Weld Finishing Method on Bead Surface Quality

**DOI:** 10.3390/ma16145100

**Published:** 2023-07-19

**Authors:** Olha Dvirna, Agata Wieczorska, Norbert Abramczyk, Anna Lesnau

**Affiliations:** Department of Engineering Sciences, Faculty of Marine Engineering, Gdynia Maritime University, 81-87 Morska St., 81-225 Gdynia, Poland; a.wieczorska@wm.umg.edu.pl (A.W.); n.abramczyk@wm.umg.edu.pl (N.A.); a.lesnau@wm.umg.edu.pl (A.L.)

**Keywords:** post-weld finishing, surface quality, cutting tool, tooth-shaped, roughness, residual stress, X-ray diffraction

## Abstract

The article describes an innovative post-weld surface finishing method, which is characterized by moving a specialized cutting tool along a butt weld. The aforementioned method is unique for the machining allowance, which is treated as the weld bead height and is removed in one step with one pass of the cutting tool. The tool is equipped on one side with linearly arranged tooth-shaped cutting elements, with the adjacent teeth height changing and increasing according to the direction of the feed. The non-standard geometry of the cutting tool enables the finishing of a heterogeneous post-weld surface with increased hardness. The results of studying the 2D profile parameters and the 3D stereometric characteristics of the surface roughness using the optical method are presented in the article. Test samples were made of S235JR steel and butt welded with the MMA, MIG, and TIG methods. Subsequently, the welding bead was ground and finished in accordance with the innovative method to flush the bead and the base metal’s surface. Additionally, residual stress analyses were performed using the X-ray diffraction method in the surface layers of the test samples. Based on the conducted research, the influence of the innovative finishing method on the surface quality is described.

## 1. Introduction

The post-weld surface finishing method by moving the cutting tool along the weld bead is discussed in patents [1,2,3,4]. In the method, the machining allowance is treated as the weld bead height, which is flush-removed from the base material in one step during one pass of the cutting tool. The innovative tool is equipped on one side with linearly arranged tooth-shaped cutting elements, with the adjacent teeth height changing and increasing along the direction of feed. The difference in height between the first and last tooth is equal to the weld bead height. The number of teeth necessary to flush-finish the weld bead with the base metal surface depends on the difference in the first and last tooth height and how it is divided. The tooth length (understood as an extension of the flank face of a tooth in an axial direction of the cutting tool) is greater than half the distance between the adjacent cutting teeth, which enables the finishing of the heterogeneous post-weld surfaces with many defects and increased degrees of hardness. The penultimate and last tooth in this invention presents the same height to eliminate cracks, craters, pores, and other weld defects.

The design solutions of the forming- and cutting-weld-bead methods described in modern patents cannot be used for post-weld surface finishing, although they possess similar elements [5,6,7,8,9,10]. First of all, post-weld surface finishing is not able to penetrate deep into the base material to form a groove on the weld’s surface. The weld bead can only be flush-removed from the base material. Contrarily, the welded joint loses its reliability. Therefore, the tool for post-weld surface finishing must be designed in such a way that the difference in the first and last tooth height is equal to the weld bead height, and the tooth width is equal to the maximum width of the weld bead.

As a result, a method and a corresponding tool were developed to finish welding surfaces to increase the operating durabilities of both the machine and the application parts. The one-pass cutting tool enabled the removal of the weld face allowance in a single step. In the next step, the innovative post-weld finishing technology and its influence on the surface layer quality obtained were developed and tested.

In order to conduct the planned research, a kit for clamping the multi-tooth tool for finishing post-welding surfaces was designed and manufactured. The subjects of this project were the set for clamping the multi-tooth tool to finish post-weld surfaces, the application of this set, and its production process. The tools for finishing post-weld surfaces and systems for clamping the cutting tools or abrasive tools used for machining are widely known. A system for universal tool clamping in a multifunctional grinding machine was discussed in the description of the patent’s application [11]. The tool clamping system was based on a movable rod cooperating with a fin positioned perpendicularly in the socket. With such an arrangement, the tool was immobilized using a screw fastener that provided a backlash-free connection between the tool and its drive unit. A chuck assembly for holding a tool in a machine tool was presented in patent description [12]. This assembly included a collet for positioning the shank portion two of the cutting tool and a lock nut for creating a clamping force between the collet and the shank portion of the cutting tool. Patent applications [1,2,3], by contrast, depict a multi-tooth tool for finishing post-weld surfaces made in the form of a steel rod of a rectangular cross-section provided on one of the external surfaces with cutting elements in the form of teeth, with the teeth arranged linearly, and their number depending on the length of the weld to be machined; however, there was no mounting of such a tool.

Thus far, no such solution has been recognized that would allow the use of a standard machine, especially a hydraulic machine designed for surface cutting work with a multi-tooth tool. The solutions presented above do not solve the problem of rapid setup and preparation of the machine to work with a multi-tooth tool. Additionally, they require skilled personnel to operate and often do not maintain the required tolerance. Moreover, a serious problem is generated by the anchoring of tools in the machine, which requires additional tools or supporting elements in order to properly mount the cutting tool and further its correct operation. As a result, better solutions are still being sought, including those that will allow the adaptation of universal machines for machining with multi-tooth tool cutting or abrasive tools.

In order to conduct a study on the innovative post-weld finishing effect in steel specimens produced using different welding methods and its impact on the key surface quality parameters, the 2D and 3D surface roughness measurements were performed [13,14,15,16,17]. The level of selected surface roughness parameters determined its functional properties: fatigue strength, wear and corrosion resistance, contact strength, tribological properties, adhesion, and others. The problem of the influence of the roughness of the weld’s surface, after innovative machining of its face, on the fatigue strength of the joint was one of the most important technological and structural issues. We could generally say that a lower roughness contributed to an increase in strength. However, in the range of values of the roughness parameter Ra = 2.5–5 μm, the fatigue strength was more influenced by the residual stresses [18,19], their distributions after depth, the micro- and macrostructures of the surface layer, and the degree of its hardening (microhardness distribution) [20].

## 2. Materials and Methods

**Figure 1:**
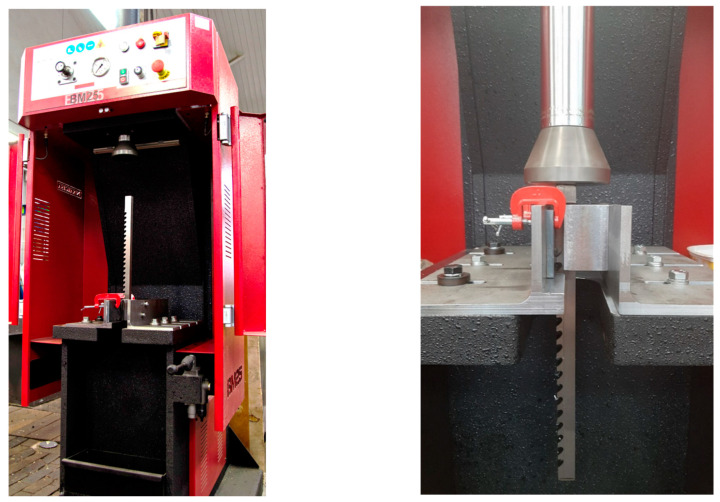
The test stand based on Nargesa’s BM25 hydraulic vertical broach.

The thesis of the process of creating the innovative method comprised the effective removal of the weld root (face) in open areas with different types of welded joints, with a guarantee of maintaining shape accuracy and high requirements to maintain the quality of the surface layer while ensuring the safety of workers and the non-obtrusiveness of the process to man and the environment. According to the stated thesis, a non-standard multi-edged tool for post-weld surface finishing was created. This cutting tool, with a specialized design adapted to specific conditions, was necessary to use for the processing of various complex surfaces of different dimensions, shapes, and qualities within one machining cycle. The multi-edged tool for post-weld surface finishing, due to the appropriate shape and cutting teeth locations, correctly adjusted to the finishing conditions on the basis of strength calculations. This tool was used in the following conditions: intermittent cuttings caused by the shapes and properties of the weld bead surfaces, uneven machining allowances, variable numbers of simultaneously working (active) edges, discontinuities of the machining process, periodically changing or impacting loads on cutting, the heterogeneity of the weld bead material, and the increase in tool wear. The Hydraulic Broaching machine BM25 NARGESA was chosen for the implementation of this non-standard cutting tool (Figure 1).

The BM25 machine was designed for small and medium production settings. This device is characterized by its great versatility, reliability, ease of use, effortless installation, effectiveness and incorporates all safety devices certified by the CE regulators. The major characteristics of the machine are as follows: motor power—2.2 Kw; 3-phased tension—230/400 V; hydraulic power—10 t; max. broaching capacity—25 mm; working speed—24 mm/s; return speed—54 mm/s; bench dimensions—420 × 420 mm.

**Figure 2:**
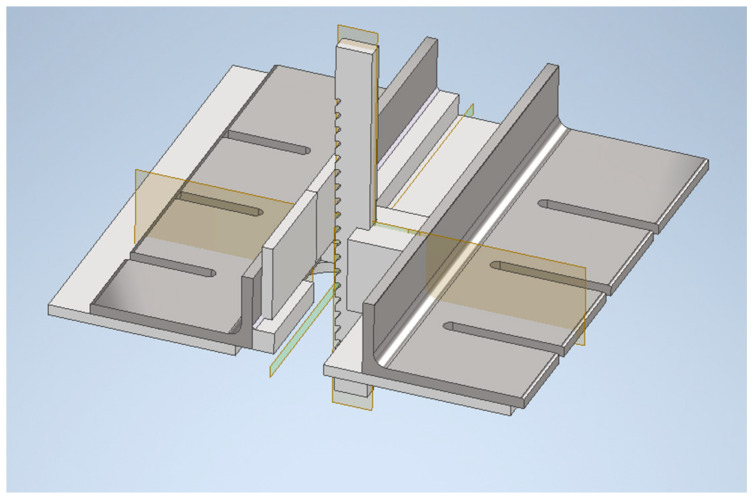
The 3D design of the tool-holding kit for butt weld finishing.

The aim of this research, among others, was to present a kit for mounting a multi-tooth tool for post-weld surface finishing. This kit enables the execution of processing and the conduction of qualitative and strength tests on the machine welded joints. To address this problem, the 3D model of the tool fixing kit was designed using the Autodesk Inventor v2022 software (Figure 2) [21].

The designed kit was utilized in a standard BM25 hydraulic machine, which was fabricated from steel and securely attached to the stationary table top of the machine. The kit comprises three structural components: the left and right angle irons made of unequal-arm steel and a stainless steel guide for the cutting tool.

**Figure 3:**
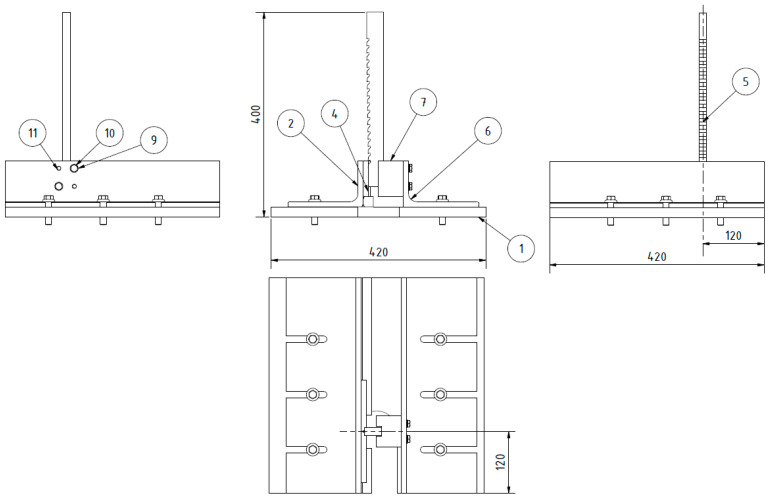
The design elements of the tool-holding kit for butt weld finishing.

**Figure 4:**
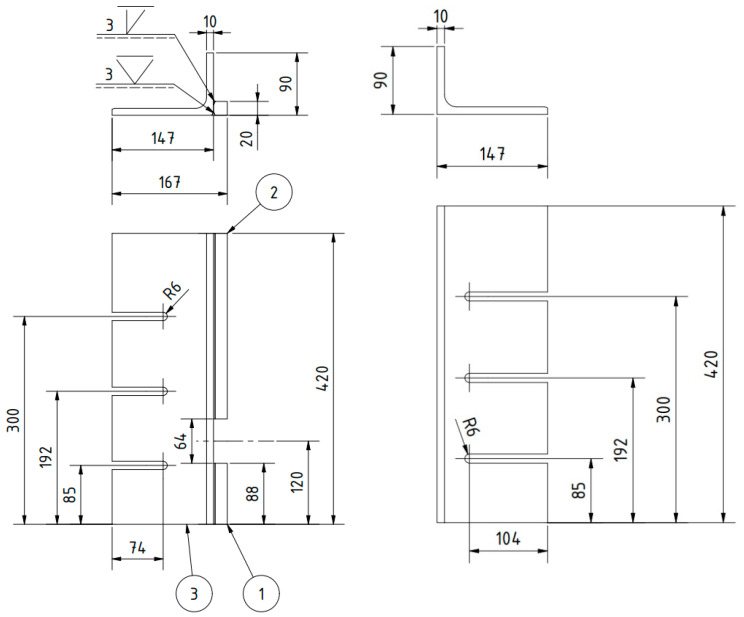
The dimensions of the left and right unequal steel angles.

The subject of the project is illustrated in the manufacturing example presented in Figure 3 and Figure 4. The elements and components are identified as follows: 1—top of the machine table, 2—design structural element I (a left unequal-arm steel angle bar), 3—hot-rolled steel profile (longer arm of the angle bar), 4—square steel bar (shelf for holding workpieces), 5—multi-tooth cutting tool, 6—design element II (right angle steel unequal arm), 7—design element III (guide with a slot of the width of the multi-tooth cutting tool to guide and support it), 8—M8 threaded holes, 9—washer, 10—M8 hexagonal head screw, 11—rolling pin.

The outer side of the left angle iron was equipped with a shelf designed to secure the machined specimens of the butt welded joints. This shelf was positioned vertically, opposite the multi-tooth tool, to ensure that the last tooth of the tool effectively removed the entire machining allowance (i.e., the height of the weld bead) without excessively cutting into the specimen's surface. The shelf was constructed using a 20 × 20 mm square steel bar, with a 64 mm gap provided for the tool's exit (Figure 4).

**Figure 5:**
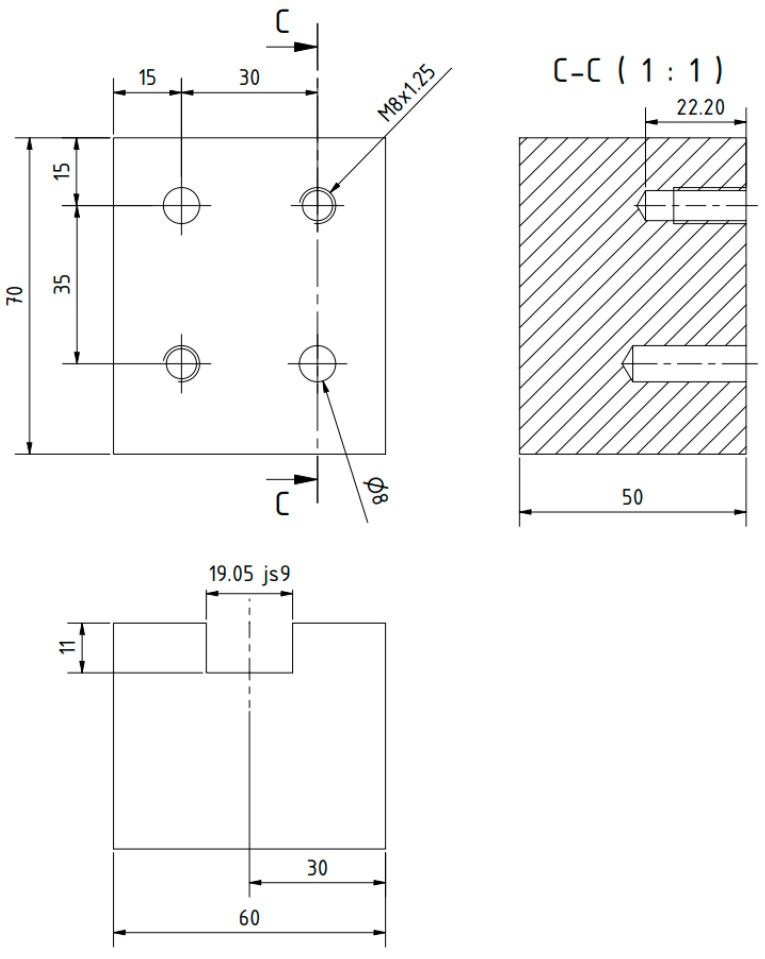
The guide for the multi-tooth tool.

On the other hand, the right angle iron was equipped with guides featuring a centrally located groove. These guides facilitated the vertical (cutting) movement of the innovative cutting tool. The groove dimensions were 14.28 ± 0.09 mm and 19.05 ± 0.09 mm in width. The guide itself was made of stainless steel with enhanced hardness. It was mounted on the outer side of the angle iron using two 9 ± 0.05 mm pins and two M8 screws, as depicted in Figure 5. 

**Figure 6:**
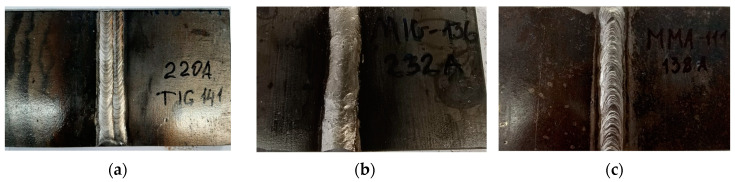
The weld-joint samples after applying different welding methods: (**a**) TIG; (**b**) MIG; (**c**) MMA.

A series of welded joints were fabricated using different welding methods (MMA, MIG, TIG) on S235JR steel to investigate the impact of the innovative post-weld finishing method on the quality of bead surfaces. Figure 6 illustrates the selected samples that were specifically prepared for testing purposes. 

After welding, the specimens were subjected to an NDT [22,23] magnetic particle inspection to reveal welding defects. Subsequently, the weld face of the samples was removed according to the innovative post-weld surface treatment method. In order to visually analyze the obtained surface and determine the areas of characteristic roughness, images were taken with a magnification of 32 times on a Zeiss SmartZoom 5 digital industrial microscope.

In order to conduct research related to the analysis of the impact of an innovative method of finishing (removal) on butt weld faces in steel specimens and its effect on the main parameters of the geometric structure of the surface, measurements of surface roughness were performed using a non-contact optical method. Additionally, visual representation of the roughness and surface topography of the selected area was obtained using an Alicona Infinite Focus G6 [24,25,26,27,28]. Both the 2D profile parameters (Ra, Rz, Rt, Rv, Rz) and thestereometric characteristics of the 3D surface roughness (Sq, Sp, Sv, Sz, Sa) determining its functional properties were tested.

**Figure 7:**
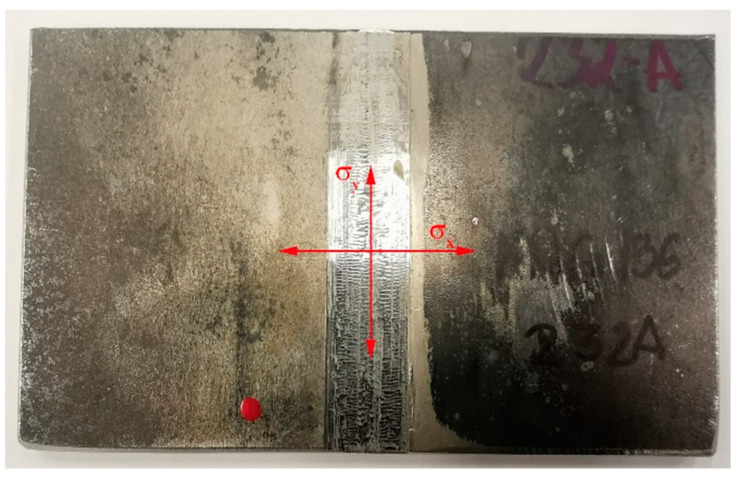
The directions of residual stress measurement σ_x_ and σ_y_ in the surface layers of the tested samples.

In the next stage of the study, the welded samples were subjected to an X-ray diffraction analysis to determine the values of residual stresses in the specified areas, both parallel and perpendicular to the direction of welding as illustrated in Figure 7. 

The residual stress was determined using a ProtoiXRD Combo diffractometer manufactured by Proto Manufacturing. The sin2Ψ method [29,30] was employed to examine the values of residual stresses. For the conducted tests, characteristic radiation of CrKα with a beam diameter of 2 mm was utilized. The anode voltage and anode current were set at 20 kV and 4 mA, respectively.

## 3. Results and Discussion

**Figure 8:**
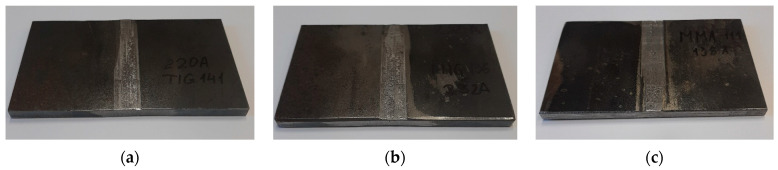
The weld-joint samples after finishing according to the innovative method: (**a**) TIG; (**b**) MIG; (**c**) MMA.

**Figure 9:**
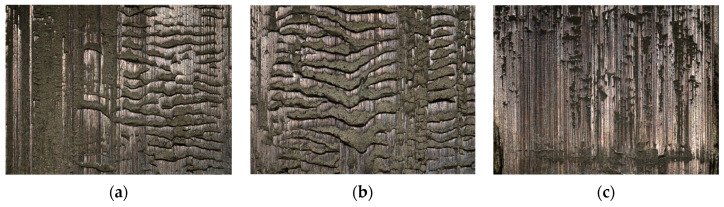
The weld-joint surface after finishing according to the innovative method with 32× magnification: (**a**) TIG; (**b**) MIG; (**c**) MMA.

The experimental weld joint samples after the innovative post-welding finishing are depicted in Figure 8. Additionally, Figure 9 presents images with a magnification of 32 times.

**Figure 10:**
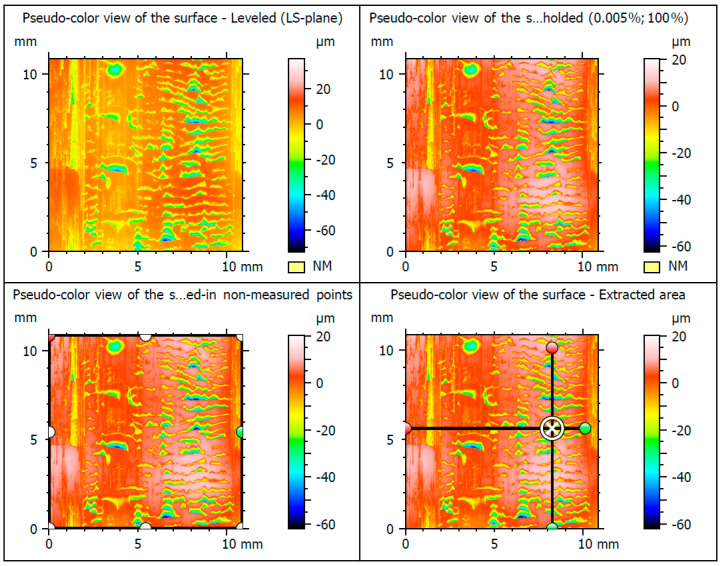
The surface of the test samples welded using the TIG.

**Figure 11:**
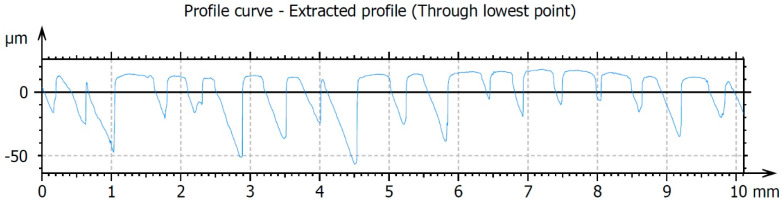
The surface roughness profile of the TIG-welded specimens after finishing was measured in the Y-axis direction, which corresponds to the direction along the weld (i.e., the direction of cutting).

**Figure 12:**
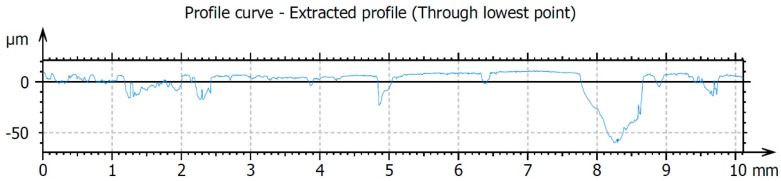
The surface roughness profile of the TIG-welded specimens after finishing was measured in the X-axis direction, which corresponds to the direction across the weld (i.e., perpendicularly to the cutting direction).

The results of testing the surface unevenness of the weld obtained with TIG welding, after removing the face using the innovative method, are presented in Figure 10 and in the graphs shown in Figure 11 and Figure 12. The measurements of the profile unevenness in the specified area were conducted in both the Y-axis (vertical) and the X-axis (horizontal) directions, considering measurements from the lowest point and from the surface. The detailed results of these measurements are summarized in Table 1.

The surface roughness measured in the horizontal X-axis direction is Ra = 8.69 μm, which is lower than the roughness value measured in the vertical Y-axis direction, which is Ra = 13.4 μm. This difference is attributed to the accuracy of the cutting edge finish, which copies the profile of the tool edge on the work surface in the X-axis direction, as well as the kinetics of the cutting process. The cutting kinetics of the innovative post-weld finishing method are based on a simple kinematic cutting scheme, where the main movement is rectilinear and directed along the cutting speed (vertically downward along the Y-axis). In the study of roughness on the weld surface after machining, the kinematics have a greater influence on the roughness level in the Y-axis direction.

**Figure 13:**
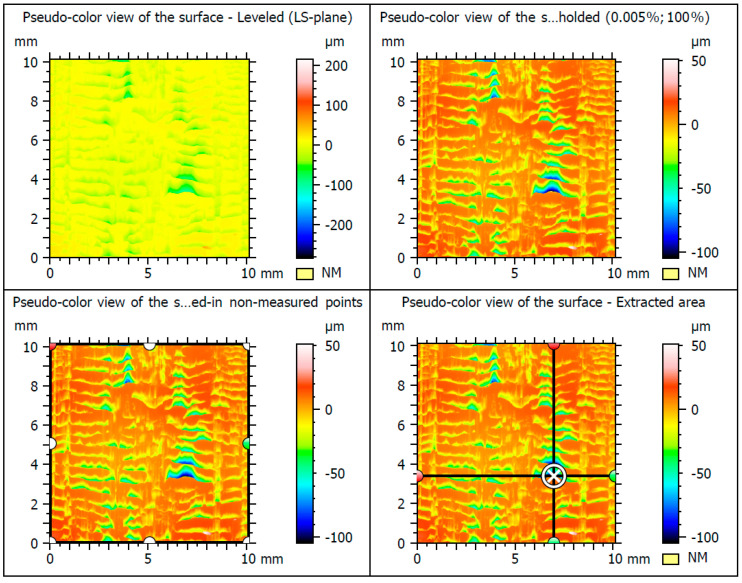
The surface roughness parameters of the MIG-welded after finishing.

The results of surface irregularities of the MIG welded joint surface after machining are illustrated in Figure 13 and are also included in Table 1.

For the MIG-welded specimens, the tests of the main roughness parameters Ra in the X and Y-axis directions were similar, at 16.2 μm and 17.0 μm, respectively. However, the height parameters were slightly smaller in the X-axis direction.

**Figure 14:**
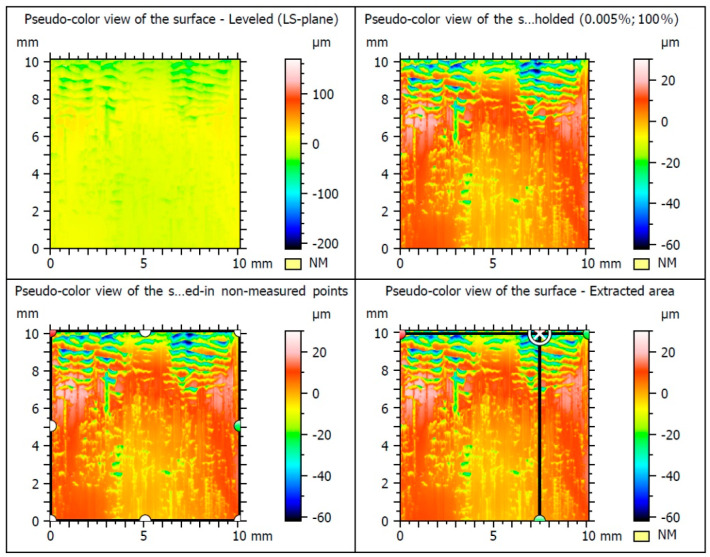
The surface roughness parameters of the MMA-welded after finishing.

In Figure 14 and in Table 1, the results of surface roughness measurements of welded joints using the MMA method after the application of the innovative post-weld finishing method are shown.

For these samples, a higher roughness value of Ra = 9.19 μm was measured in the X-axis direction, while in the Y-axis direction, it was only Ra = 7.02 μm. Conversely, in the measurements of samples after TIG welding, a lower roughness level was observed. Most probably, this is related to the method of standing and the properties of the filler metal, rather than to their processing.

Comparing the results of all the TIG, MIG, and MMA welded specimens machined according to the innovative method, it was found that the lowest roughness value of Ra = 7.02 μm was obtained when measuring the MMA weld face along the Y-axis. Conversely, the highest Ra = 17.0 μm was measured on a sample of a welded joint using the MIG method in the Y-axis direction.

The total height of the Rt profile varied for different samples, ranging from 38.2 μm (TIG) to 65.0 μm for samples welded using the MMA method (Table 2).

The influence of the amplitude parameters of surface roughness, such as Ra (Rz) and Rt, on fatigue strength was found to be critical. This is because the depth of the profile grooves served as an indicator of stress concentration.

The results of recording three-dimensional metrological measurements of the surface were tabulated. The surface topography was characterized by the following parameters: Sq, Sp, Sv, Sz, Sa. Root mean square height, or root mean square deviation of the surface (Sq), was defined analogously to Rq and calculated as the standard deviation of the height of surface irregularities with respect to the reference surface. The highest value of Sq = 13.9 μm was obtained when the sample was measured after the MIG welding. In contrast, the smallest Sq = 9.21 μm was measured when testing the TIG samples. A remarkably similar result of Sq = 9.24 μm was obtained when measuring samples welded using the MMA method (Table 3).

The next measured height parameters were recorded—the height of the highest surface elevation Sp (maximum peak height) and the depth of the lowest pit Sv (maximum pit depth). The highest levels of these parameters were observed in tests of the MIG-welded specimens, with Sp = 44.2 μm and Sv = 105 μm. Similarly, an analogous occurrence appeared during the study of the maximum surface height Sz (maximum height) and the arithmetic mean surface height Sa (arithmetic mean height). The highest values of Sz = 149 μm and Sa = 10.3 μm were observed in the MIG specimens (Table 3).

In order to determine the influence of the innovative finishing method the weld bead obtained according to different welding methods on the surface quality, the study of the residual stresses before and after finishing was performed [29,30]. It should be remembered that in the range of roughness parameter Ra above 5 μm, which was tested in this study, the decisive influence on fatigue strength was not the geometric parameters of the surface layer quality, but the physicalmechanical and structural ones, such as the residual stresses, their distribution by depth, micro and macrostructure of the surface layer and microhardness.

**Figure 15:**
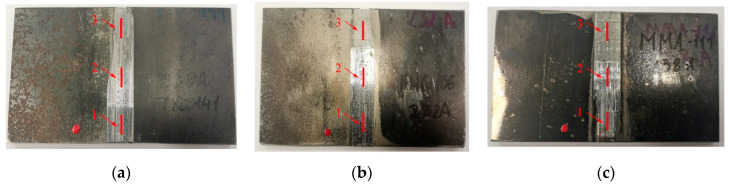
The samples prepared for X-ray diffraction residual stress tests with marked areas of measurement: (**a**) TIG; (**b**) MIG; (**c**) MMA.

Figure 15 shows the specimens with three designated areas (1,2,3) where measurements were conducted. 

The values of residual stresses were measured in the designated areas along two directions, namely the X-axis and the Y-axis. The specific conditions of the measurements are provided in Table 4.

The measurement with the standard deviation are listed in Table 5 and Table 6.

Compressive residual stresses were present on the surfaces of all the samples (TIG, MIG, MMA) both before and after treatment using the innovative post-weld finishing method. 

The highest level of compressive residual stresses in the specimens before finishing was observed in the specimens welded using the MIG method in the horizontal X-axis direction, with a value of σ_x_ = −347 MPa. Similarly, after welding using the MMA method, a value of σ_x_ = −205 MPa was measured for compressive residual stresses in the same direction. In the specimens after innovative finishing method, the highest compressive residual stresses were observed on the surface of the MMA specimen, with a value of σ_x_ = −483 MPa. The smallest value of σ_x_ = −12 MPa was observed in tests of the sample after the MIG welding.

## 4. Conclusions

The custom-designed and manufactured set for mounting a multi-tooth tool for finishing post-weld surfaces and butt welded joints enabled the successful implementation of the planned research on the impact of the innovative post-weld finishing method on bead surface quality.

The main advantages of the presented solution were the speed and ease of the assembly of the multi-tooth tool, which facilitated precise work. The tool enabled a vertical, reciprocating, and stable movement along the weld, effectively removing the weld face allowance.

One of the positive aspects of the proposed solution was the ability to quickly configure and prepare the machine to perform operations on welds of different widths and using various welding methods such as MMA, MIG, and TIG. This flexibility allowed efficient utilization of the machine across a range of welding applications. The machine equipped with the tool-holding kit was designed for easy operation, eliminating the need for handling by qualified personnel. The use of the kit ensured both speed and precision in the processing operations while also minimizing or eliminating environmental pollution. The proper attachment of the kit to the table of the hydraulic machine tool ensured precise movement of the tool along the stitch of welded joints with different shapes and dimensions. The tool attachment kit allowed the tool be guided with respect to the vertical axis perpendicularly to the surface of the machine tool table effectively and without the use of the operator’s force, removing the excess face. Chips of the removed surplus fell freely into the vessel under the table on which the kit was mounted, without clogging the tool used.

The machining process resulted in the formation of a high-quality surface layer with excellent mechanical properties, as demonstrated by the conducted tests. Based on the analysis of the results obtained from the study of surface profile unevenness and topography, it can be concluded that the innovative post-weld finishing method can be applied to steel butt welded joints using the TIG, MIG, and MMA welding methods without reducing the surface quality and fatigue strength of the joint.

The use of the innovative method enabled the generation of favorable compressive residual stresses of up to −483 MPa on the surface of the treated weld for a joint welded using the MMA method. This represents a significant increase of more than 50% compared to the pre-treatment value, which did not exceed −205 MPa. This increase in compressive stress significantly enhances the strength and reliability of the joint. 

The obtained test results accurately reflect the theoretical basis for the formation of second-kind residual stresses in the surface layer after post-weld finishing in combined with burnishing. These results can be effectively applied in various studies related to the formation of technological quality in the surface layer of steel components.

## Figures and Tables

**Table 1:** The roughness measurement results of the extracted profile through the lowest point in the direction of the Y and X-axis (ISO 21920).

Roughness Parameters [μm]	TIG Sample		MIG Sample		MMA Sample	
	Y-Axis	X-Axis	Y-Axis	X-Axis	Y-Axis	X-Axis
Rz	60.6	35.7	75.3	51.3	37.8	45.6
Ra	13.4	8.69	17.0	16.2	7.02	9.19

**Table 2:** The roughness measurement results of the series of profiles taken from the surface in the Y and X-axis directions (ISO 21920).

Roughness Parameters [μm]	TIG Sample		MIG Sample		MMA Sample	
	Y-Axis	X-Axis	Y-Axis	X-Axis	Y-Axis	X-Axis
Rt	38.2	43.6	65.0	64.1	46.6	37.9
Rv	21.7	9.96	33.2	13.7	18.7	9.83
Rz	27.9	15.6	45.0	22.7	26.8	16.0
Ra	5.45	3.88	9.19	5.34	5.16	3.36
Rc	20.5	17.3	32.6	25.8	22.3	14.0

**Table 3:** The results of the 3D surface measurements of the extracted area in the tested weld-joint samples (ISO 25178).

Roughness Parameters [μm]	TIG Sample	MIG Sample	MMA Sample
Sq	9.21	13.9	9.24
Sp	26.1	44.2	24.5
Sv	64.7	105.0	57.1
Sz	90.9	149.0	81.5
Sa	6.55	10.3	6.53

**Table 4:** The conditions for measuring the residual stresses in the surface layers of the samples.

Parameter	Value
Material	S235JR
Depth, µm	0
Young’s modulus, GPa	210
Poisson’s number	0.3
Value of constants 1/2S2 and -S1	1/2S2 = 6.19 × 10^−6^ MPa; −S1 = 1.42 × 10^−6^ MPa
Crystallographic plane (hkl)	(211)
Bragg’s angle (2θ), degree	156.40
X-ray lamp	Cr Kα λ = 0.2291 nm
Accelerating voltage and current	20 kV; 4 mA
Background profile (Gain material)	Ti-β
Gain power	17 kV; 4 mA
Filter	Lack
Collimator	Round; 2 mm
Goniometer configuration	ψ, two detectors
The number of angles β/ψ	11
Angle ψ, degree:	±13.20; ±8.77; ±3.99; ±8.19; ±11.80; ±15.41; ±23.80; ±27.59; ±32.37; ±36.80
Oscillation	x = ±3 mm; β = ±1°
Exposure time/angle	1
The number of exposures	10

**Table 5:** The results of the residual stress analysis by X-ray diffraction on the weld-joint surface (TIG, MIG, MMA) before finishing.

Residual Stress Value [MPa]	TIG Sample	MIG Sample	MMA Sample
σ_x_	−309	−347	−205
σ_y_	−197	−230	−142

**Table 6:** The results of the residual stress analysis by X-ray diffraction on the weld-joint surface (TIG, MIG, MMA) after the application of the innovative post-weld finishing method.

Residual Stress Value [MPa]		TIG Sample	MIG Sample	MMA Sample
σ_x_	area 1	−411	−325	−483
	area 2	−404	−315	−66
	area 3	−450	−12	−19
σ_y_	area 1	−429	−320	−378
	area 2	−407	−263	−41
	area 3	−408	−32	−57

## Data Availability

The data presented in this study are available on request from the corresponding author. The data are not publicly available due to privacy restrictions.
